# Anatomical attention can help to segment the dilated pancreatic duct in abdominal CT

**DOI:** 10.1007/s11548-023-03049-z

**Published:** 2024-03-18

**Authors:** Chen Shen, Holger R. Roth, Yuichiro Hayashi, Masahiro Oda, Gen Sato, Tadaaki Miyamoto, Daniel Rueckert, Kensaku Mori

**Affiliations:** 1 Graduate School of Informatics, Nagoya University, Furo-cho, Nagoya, Aichi 4648601 Japan; 2 NVIDIA Corporation, San Tomas Expy, Santa Clara, CA 95051 USA; 3 Information Strategy Office, Information and Communications, Nagoya University, Furo-cho, Nagoya, Aichi 4648601 Japan; 4 Chiba Kensei Hospital, Makuhari-cho, Chiba, Chiba 2620032 Japan; 5 Department of Computing, Imperial College London, Exhibition Road, London, SW7 2AZ UK; 6 Klinikum rechts der lsar, Technical University of Munich, Ismaninger Str. 22, Munich, 81675 Germany; 7 Research Center for Medical Bigdata, National Institute of Informatics, 2-1-2 Hitotsubashi, Tokyo, 1018430 Japan

**Keywords:** Dilated pancreatic duct, Pancreatic duct segmentation, Anatomical attention, Tubular structure enhancement

## Abstract

**Purpose:**

Pancreatic duct dilation is associated with an increased risk of pancreatic cancer, the most lethal malignancy with the lowest 5-year relative survival rate. Automatic segmentation of the dilated pancreatic duct from contrast-enhanced CT scans would facilitate early diagnosis. However, pancreatic duct segmentation poses challenges due to its small anatomical structure and poor contrast in abdominal CT. In this work, we investigate an anatomical attention strategy to address this issue.

**Methods:**

Our proposed anatomical attention strategy consists of two steps: pancreas localization and pancreatic duct segmentation. The coarse pancreatic mask segmentation is used to guide the fully convolutional networks (FCNs) to concentrate on the pancreas’ anatomy and disregard unnecessary features. We further apply a multi-scale aggregation scheme to leverage the information from different scales. Moreover, we integrate the tubular structure enhancement as an additional input channel of FCN.

**Results:**

We performed extensive experiments on 30 cases of contrast-enhanced abdominal CT volumes. To evaluate the pancreatic duct segmentation performance, we employed four measurements, including the Dice similarity coefficient (DSC), sensitivity, normalized surface distance, and 95 percentile Hausdorff distance. The average DSC achieves 55.7%, surpassing other pancreatic duct segmentation methods on single-phase CT scans only.

**Conclusions:**

We proposed an anatomical attention-based strategy for the dilated pancreatic duct segmentation. Our proposed strategy significantly outperforms earlier approaches. The attention mechanism helps to focus on the pancreas region, while the enhancement of the tubular structure enables FCNs to capture the vessel-like structure. The proposed technique might be applied to other tube-like structure segmentation tasks within targeted anatomies.

## Introduction

**Fig. 1:**
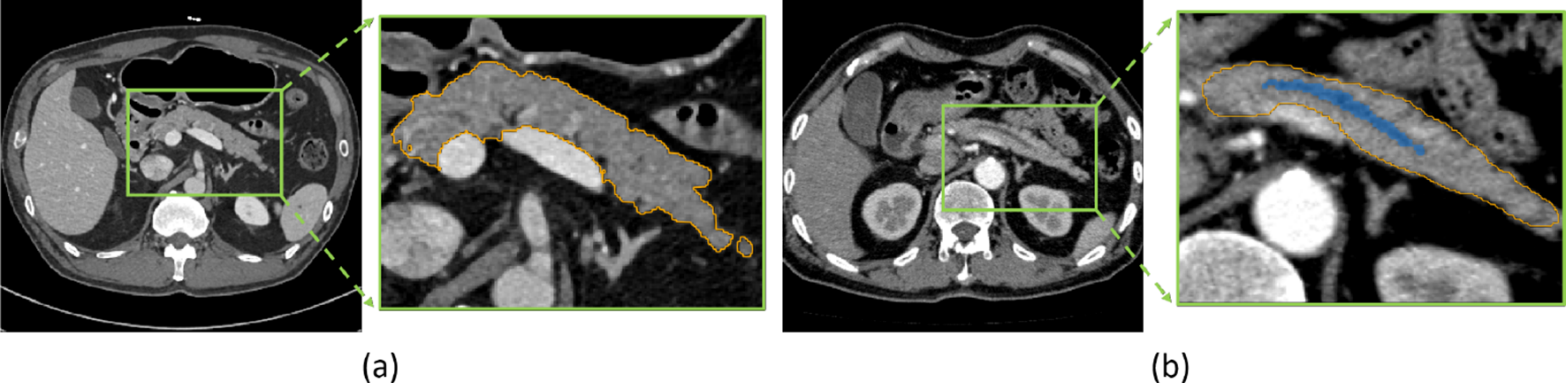
Visual examples of axial CT slices of **a** normal pancreas and **b** pancreas with dilated pancreatic duct. The pancreas region is surrounded by the orange contour, and the pancreatic duct is indicated by blue

Pancreatic cancer is one of the most deadly malignancies, killing hundreds of thousands of people every year around the world. Compared to other malignancies, it exhibits the lowest 5-year survival rate, which is approximately 10% in the USA [1]. Due to the mild symptoms, pancreatic cancer is difficult to detect until it has reached an advanced stage. Pancreatic ductal adenocarcinoma (PDAC), which develops in the main duct, accounts for more than 90% of pancreatic cancer [2]. Several clinical studies suggest that dilatation of the main pancreatic duct indicates an increased risk of pancreatic cancer [3, 4]. Therefore, the appearance of pancreatic duct dilatation may serve as a useful entry point for diagnosing pancreatic cancer. However, the main duct of a healthy pancreas is not apparent on the CT scans, as seen in Fig. 1a. On the other hand, if the main pancreatic duct is dilated, a dark line structure can be observed inside the pancreas region as shown in Fig. 1b. Due to this fact, we expect that the automated segmentation of dilated pancreatic ducts from CT volumes could aid in the early detection of pancreatic cancer.

In the past few years, several articles have been devoted to the study of PDAC segmentation  [5–9] and its surrounding anatomy such as blood vessels potentially useful for evaluation of treatment response [9], but excluded the automated segmentation of the pancreatic duct itself. However, only a few consider the pancreatic duct region a discrete segmentation target [6, 7]. Both Zhou et al. [6] and Xia et al. [7] investigated pancreatic duct segmentation methods on a large number of precisely annotated venous and arterial phase CT volumes, which are extremely difficult to obtain. All CT volumes are from patients who have already been diagnosed with PDAC. Only a few attempts have so far been made on pancreatic duct segmentation in persons without any type of pre-existing pancreatic cancer. Shen et al. [10] presented a cascade framework for dilated pancreatic duct segmentation using single-phase CT volumes. The region of the pancreas was cut off, according to the segmentation of the pancreas, and only the pancreas regions were utilized as inputs for the pancreatic duct segmentation.

**Table 1:** The GPU memory requirements for different input sizes when using the standard 3D U-Net and V-Net are listed

Network	Input size	Parameters #	GPU memory usage
3D U-Net [19]	$128^3$	19.1 M	$\sim$20 GB
V-Net [20]	$128^3$	41.2 M	$\sim$5.8 GB
3D U-Net	$160^3$	19.1 M	$\varvec{>}$**24 GB**
V-Net	$160^3$	41.2 M	$\sim$9.7 GB

The batch size is 2 in all the following settings

The pancreatic duct segmentation from patients who have not yet developed PDAC is difficult. The primary challenge in dilated pancreatic duct segmentation is the tiny size of the pancreatic duct region in comparison with the whole abdominal CT volume. After developing PDAC, the duct regions would be significantly thicker than that of a healthy person. Fully convolutional networks (FCNs) are the principal method for semantic segmentation, which effectively reduces the complexity of this task. However, some limitations result in the segmentation performance being unacceptable when dealing with small targets. The significant bias between the number of foreground and background voxels will affect the segmentation performance when utilizing FCNs. To overcome this issue, we propose a framework for dilated pancreatic duct segmentation based on anatomical attention. In deep learning-based methods, the attention mechanism is typically used to boost the influence of pertinent information and reduce irrelevant context. In medical image analysis, numerous attention strategies are applied and successfully improve FCN performance [11–13]. We assume that allowing the FCN to concentrate on the pancreas region during training will be beneficial for the segmentation of the dilated pancreatic duct. To achieve this, we propose a pancreatic anatomical attention-based method inspired by the Attention U-Net architecture [12]. To fully utilize the information produced at different scales, we employ the multi-scale aggregation before the final prediction. Considering that the anatomical structure of the pancreatic duct is similar to blood vessels, incorporating the tubular features during training may further help the FCN understand the duct’s connection component. We incorporated the feature information gained from the tubular structure enhancement filter [14] as an additional input of our FCN.

This work is an extension of our earlier report [15] at a scientific conference, which was included in the conference proceedings published 10th Workshop, CLIP 2021 of MICCAI 2021. We improved the following four aspects in this journal: (1) enhanced the FCNs for coarse pancreatic segmentation to fully raise network performance with limited computer power; (2) introduced tubular structure enhancement as an extra FCN input for better learning anatomical features; (3) greatly improved segmentation accuracy on dilated pancreatic duct; (4) more metrics were employed to evaluate the efficacy of our proposed strategy.

## Methods

### Overview

The framework we propose consists of two main steps. Firstly, we develop a straightforward but efficient pancreatic mask segmentation model using the publicly available pancreas dataset [16]. This model can be used to produce the coarse pancreas mask for the dilated pancreatic duct dataset. Then, we crop the pancreas ROIs based on the masks and only use the ROIs for pancreatic duct segmentation. The pancreas mask obtained from the first step can further utilized to guide the FCN for anatomical attention. Additionally, we incorporate a tubular structure enhancement as an additional input channel for FCN.

### Coarse pancreatic mask segmentation

Since the pancreatic duct only takes up a small portion of the abdominal CT volume, it is particularly challenging to segment the target directly. Our preceding study suggests that using the pancreas ROIs in FCN during the dilated pancreatic duct segmentation will help train the FCN to concentrate on the pancreatic region [10]. In this study, we develop a straightforward yet effective pancreatic mask segmentation model using the publicly accessible dataset [17] for coarse pancreas mask segmentation. U-Net [18] is a ready-to-use FCN that has proven to be useful in the field of medical image segmentation. 3D U-Net [19] and V-Net [20] are well-known 3D extensions for U-Net, which show considerable power in handling 3D volumes instead of 2D images. As shown in Table 1, the standard V-Net can hold larger input volume sizes with less memory usage than the standard 3D U-Net. Due to this fact, we use V-Net as our baseline for the coarse pancreatic mask segmentation.

Scaling up the networks in depth, width, and resolution aspects has been shown to be beneficial in boosting segmentation performance [21]. When it comes to the V-Net, increasing the number of resolution levels of the network helps it to capture more specific information from the input volumes. Extending the input size of FCNs can also benefit the network by allowing it to handle larger volumes and more detailed contexts. However, simply scaling up a neural network is not always the best approach because larger networks are more computationally expensive to train and may be more prone to overfitting. Model complexity must be carefully balanced with computational efficiency and generalization performance. We scale up the standard V-Net to discover the most efficient type for coarse pancreas segmentation and then utilize it as a baseline for pancreatic duct segmentation in this work. We further introduced different types of normalization techniques, including batch normalization (BN) and instance normalization (IN).

### Anatomical attention-based duct segmentation

#### Network architecture

**Fig. 2:**
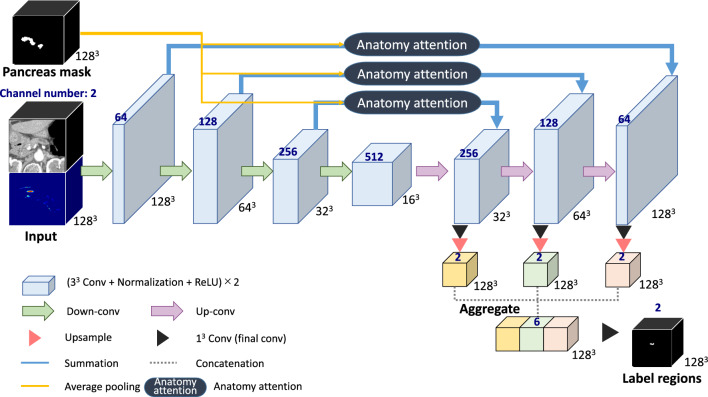
The proposed anatomical attention FCN architecture. The channel numbers are listed above the boxes in blue. The convolution blocks are shown by the blue boxes, and the feature map sizes are indicated next to the boxes

For the dilated pancreatic duct segmentation, we proposed an anatomical attention-based FCN as shown in Fig. 2. The FCN structure consists of an encoder part and a decoder part with resolution levels of four. A training set $\textbf{S}=\{\textbf{I}_n,\textbf{L}_n,\textbf{M}_n,\textbf{P}_n, n=1,\ldots ,N\}$ is prepared, where $\textbf{I}_n\in \mathcal {R}^{W\times H\times D}$ indicates the *n*-th CT volume from the total *N* training sets. The volume size in width, height, and depth of the *n*-th training set is $W\times H\times D$. $\textbf{L}_n$ represents the corresponding ground-truth volume of the pancreatic duct region, and $\textbf{M}_n$ represents the mask of the pancreas region, which is from the segmentation result in “Coarse pancreatic mask segmentation” section. $\textbf{P}_n$ is the features generated from the tubular structure enhancement filter. The input of our FCN is a two-channel union of CT volume $\textbf{I}_n$ and the corresponding pancreatic duct enhancement $\textbf{P}_n$. The coarse pancreas prediction mask $\textbf{M}_n$ is employed to guide the anatomical attention on each level for the decoder. In multi-level FCNs, the high-resolution features are typically more focused on spatial information, whereas low-resolution features usually concentrate on the semantic information from the input. Combining features from multiple scales enables the learning of additional complementary information, which helps boost and refine the final prediction [13, 22]. Therefore, we aggregate the feature maps from each level to produce the final segmentation similar to the deep supervision [23].

#### Anatomical attention

**Fig. 3:**
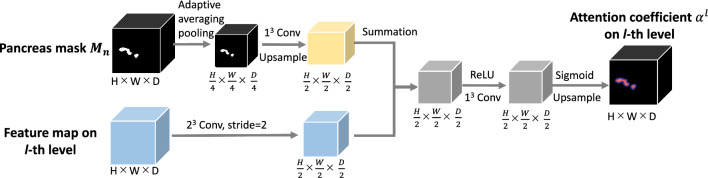
The process of computing the anatomical attention coefficient on *l*-th level of FCN. The colored boxes are denoting the feature maps, and the feature map sizes are indicated under the boxes

Our anatomical attention is inspired by the attention mechanism proposed for 3D U-Net, which was investigated to capture beneficial information and ignore the irrelevant context in FCNs [12]. Since the dilated pancreatic duct only makes up a small fraction of the entire pancreas, we prefer to focus on the whole pancreas rather than just the target. An attention coefficient $\mathcal {A}_j^l\in [0,1]$ can be computed for each level *l* of the FCN based on the grid attention [11], where *j* is the *j*-th voxel of the input image. The coarse pancreas segmentation is used as a mask for the attention gate to provide spatial information. The detailed procedure of calculating the attention coefficient vector ${\varvec{\mathcal {A}}}^l$ on the *l*-th level is shown in Fig. 3. The pancreas masks $\textbf{M}_n$ are downsampled using an adaptive averaging pooling to match the size of the bottleneck layer and are followed by a $1\times 1\times 1$ convolution to learn the pixel-wise focus regions $\textbf{g}_n$. For the input feature map, apply a $2\times 2\times 2$ convolution with stride 2. Then sum up the output with the focus region $\textbf{g}_n$ over the channel dimension. An attention vector ${\varvec{\mathcal {A}}}^l$ with values between 0 and 1 is produced by the sigmoid activation function. Furthermore, upsample operations are necessary to make the attention vector’s size suitable for each level’s feature map. The output of anatomical attention is obtained by multiplying the input feature map $\textbf{x}^l$ and attention vector elementally, as

1 $$\begin{aligned} \hat{\textbf{x}^l} = {\varvec{\mathcal {A}}}^l \cdot \textbf{x}^l. \end{aligned}$$

#### The tubular structure enhancement channel

To fully capture the tubular structure of the pancreatic duct, we introduce an additional channel as input to our FCN. In vessel segmentation, vessel enhancement algorithms are often incorporated to increase the robustness of segmentation performance [24]. Most of these algorithms try to depict the curvature of the vessel-like structure with the second derivatives of the volume intensities. In the medical image analysis field, the Frangi filter is commonly used as the vessels enhancement filter to identify tubular structures and suppress other image features such as noise and non-vessel structures [14]. It is a Hessian-based method proposed to strengthen the differences in intensity in medical volumes with eigenvalues $|\lambda _{1}| \le |\lambda _{2}| \le |\lambda _{3}|$, where $\lambda _{1}, \lambda _{2}, \lambda _{3}$ are derived from the Hessian matrix to indicate the principal curvatures of the intensity profile at each voxel. An ideal tubular structure in 3D voxel has $|\lambda _1|\approx 0,|\lambda _1|\ll |\lambda _2|,\lambda _2\approx \lambda _3$. The Frangi filter is formulated as:

2 $$\begin{aligned} F{=} {\left\{ \begin{array}{ll} \left( 1{-}\exp \left( {{-}\frac{R^2_a}{2\alpha ^2}}\right) \right) \exp \left( {-}\frac{R^2_b}{2\beta ^2}\right) &{} \\ \left( 1{-}\exp \left( {-}\frac{S^2}{2\gamma ^2}\right) \right) , &{} \text {if }\lambda _2{\le }0\text { and }\lambda _3{\le }0, \\ 0, &{}\text {otherwise}, \\ \end{array}\right. }\nonumber \\ \end{aligned}$$

where $\alpha =0.5$ and $\beta =0.5$ are fixed by experience to control the sensitivity of the filter, and $\gamma$ uses half of the maximum Hessian norm of the intensity range [14, 25]. $R_a$ is used to distinguish the tubular-like and the plate-like structure, $R_b$ to measure the blob-like structure, and *S* indicates the low-contrast backgrounds. These patterns can be formulated as:

3 $$\begin{aligned} R_a=\frac{|\lambda _2|}{|\lambda _3|}, R_b=\frac{|\lambda _1|}{\sqrt{|\lambda _2\lambda _3|}},~S=\sqrt{\lambda _1^2+\lambda _2^2+\lambda _3^2}. \end{aligned}$$

For post-processing, we utilize pancreatic mask segmentation to eliminate the values outside the pancreas areas. Additionally, to better adapt to the deep neural network, we apply min–max normalization to convert the remaining filter output into the range of 0 to 1.

## Experiments and results

### Experiment details

We used the publicly accessible TCIA pancreas annotation dataset [16] to develop the coarse pancreatic mask segmentation model. The dataset contains 82 contrast-enhanced portal venous phase abdomen 3D CT volumes, with $512\times 512$ pixels for each CT slice. Each CT volume has 181 to 466 slices, with slice resolution between 0.5 mm and 1.0 mm. The dataset was randomly divided into 48 training, 16 validation, and 18 testing samples. The dataset was resampled into $1\times 1$ mm isotropic resolution for the pancreatic mask segmentation. We generated the pancreas ROIs based on the segmentation results, followed by [10]. For the dilated pancreatic duct segmentation, we utilized 30 contrast-enhanced portal venous phase 3D CT volumes from persons who had the symptom of pancreatic duct dilation. We must point out that none of them have been diagnosed with PDAC. This dataset is a private dataset, with all the CT volumes taken at Chiba Kensei Hospital in Japan. The duct regions were annotated by an experienced PhD student who is knowledgeable about the pancreas and pancreatic duct dilation and its appearance in abdominal CT images. All images were manually refined slice by slice using Pluto [26]. The details of pancreatic duct annotation operations are described in [10]. Each CT slice size is $512\times 512$ pixels, and the slice numbers range from 192 to 887. The resolution of each axis is 0.59$-$0.75 mm, 0.59$-$0.75 mm, and 0.3$-$1.0 mm, respectively. The dilated pancreatic duct dataset was resampled into an isotropic resolution of $0.5\times 0.5\times 0.5$ mm. To accommodate a higher resolution, the coarse pancreatic mask was scaled up before being used in the pancreatic duct segmentation. For a fair comparison among different methods, we exclusively used cropped ROIs of the pancreas regions as input in this task. The input size of FCNs in pancreatic duct segmentation is fixed at $160\times 160\times 160$ voxels. We performed the four fold cross-validation on the pancreatic duct segmentation to ensure the reliability of the experimental results.

Our experiments are implemented on PyTorch 1.7.1, and NVIDIA Tesla V100 with 32 GB of memory is used for all experiments. For all CT volumes, we only kept the intensity within the range of [$-$ 200, 200] H.U. and then rescaled the intensity values into the range of [0, 1] with min–max normalization to better illustrate the pancreas regions. During training, we use Adam optimization with a learning rate of $10^{-4}$ to minimize the Dice loss function.

### Results

**Table 2:** An ablation study to show the effectiveness of scaling the V-Net in the network level, numbers of initial filter and input size for pancreatic mask segmentation

Level #	Initial filter #	Parameters #	Input size	Normalization	DSC (%)
4	16	9.9 M	$128^3$	BN	53.2
			$160^3$	BN	59.4
			$160^3$	IN	74.8
5	16	41.2 M	$128^3$	BN	50.9
			$160^3$	BN	67.8
			$160^3$	IN	75.7
6	16	166.2 M	$128^3$	BN	39.9
			$160^3$	BN	69.3
			$160^3$	IN	78.3
6	32	664.7 M	$128^3$	BN	44.1
			$160^3$	BN	63.3
			$160^3$	IN	**78**.**8**

The bold text indicates the best DSC

Table 2 shows the quantitative evaluation result of the coarse pancreatic mask segmentation. We used V-Net as the baseline and scaled up the network in depth, width, and resolution, which correspond to the level number, filter number, and input size. Scaling up the V-Net positively influences pancreas segmentation. In this experiment, both BN and IN were used, with IN being more beneficial for pancreas segmentation.

**Table 3:** An ablation study of using our proposed FCNs on the dilated pancreatic duct segmentation

Network	Backbone	DSC (%)	Sensitivity (%)	NSD (%)	HD95
Attention U-Net	U-Net+BN	50.7±17.0	56.8±22.3	53.1±19.4	89.5±43.6
	U-Net+IN	52.5±16.5	62.9±.21.8	59.7±.20.2	83.5±45.5
PANet	U-Net+BN	52.9±16.1	58.3±21.8	59.9± 20.2	67.8±41.1
	U-Net+IN	54.2±13.2	66.4±17.2	63.1±15.9	72.1±38.7
	V-Net+BN	52.6±17.0	62.3±22.2	60.8±19.9	72.0±42.6
	V-Net+IN	51.1±14.5	64.9±17.9	63.0±14.5	86.6±36.3
PAMNet	U-Net+BN	53.4±11.4	66.1±14.1	63.2±14.5	75.5±39.8
	U-Net+IN	52.1±13.3	**67**.**5**±**14**.**9**	63.7±15.4	77.2±46.9
	V-Net+BN	52.7±14.4	62.3±19.9	60.5±17.0	64.1±45.1
	V-Net+IN	53.8±14.1	60.7±17.4	60.8±18.4	66.9±44.9
MCPAMNet	U-Net+BN	53.3±13.1	65.2±17.5	59.9±14.0	63.0±42.4
	U-Net+IN	53.3±10.7	60.3±18.6	61.9±12.1	63.6±45.2
	V-Net+BN	53.5±13.6	65.5±17.7	60.8±16.2	66.9±43.0
	V-Net+IN	55.5±11.3	62.5±18.2	63.0±14.2	60.4±46.4
NMCPAMNet	U-Net+BN	55.2±11.9	64.4±17.2	63.2±12.2	68.4±44.1
	U-Net+IN	55.4±12.0	64.2±15.8	**63**.**7**±**13**.**4**	62.5±45.6
	V-Net+BN	55.4±13.0	62.8±19.4	63.1±14.7	65.0±44.7
	V-Net+IN	**55**.**7**±**12**.**5**	64.3±19.3	63.0±15.7	**56**.**4**±**40**.**3**

The bold text indicates the best values for each metric

**Fig. 4:**
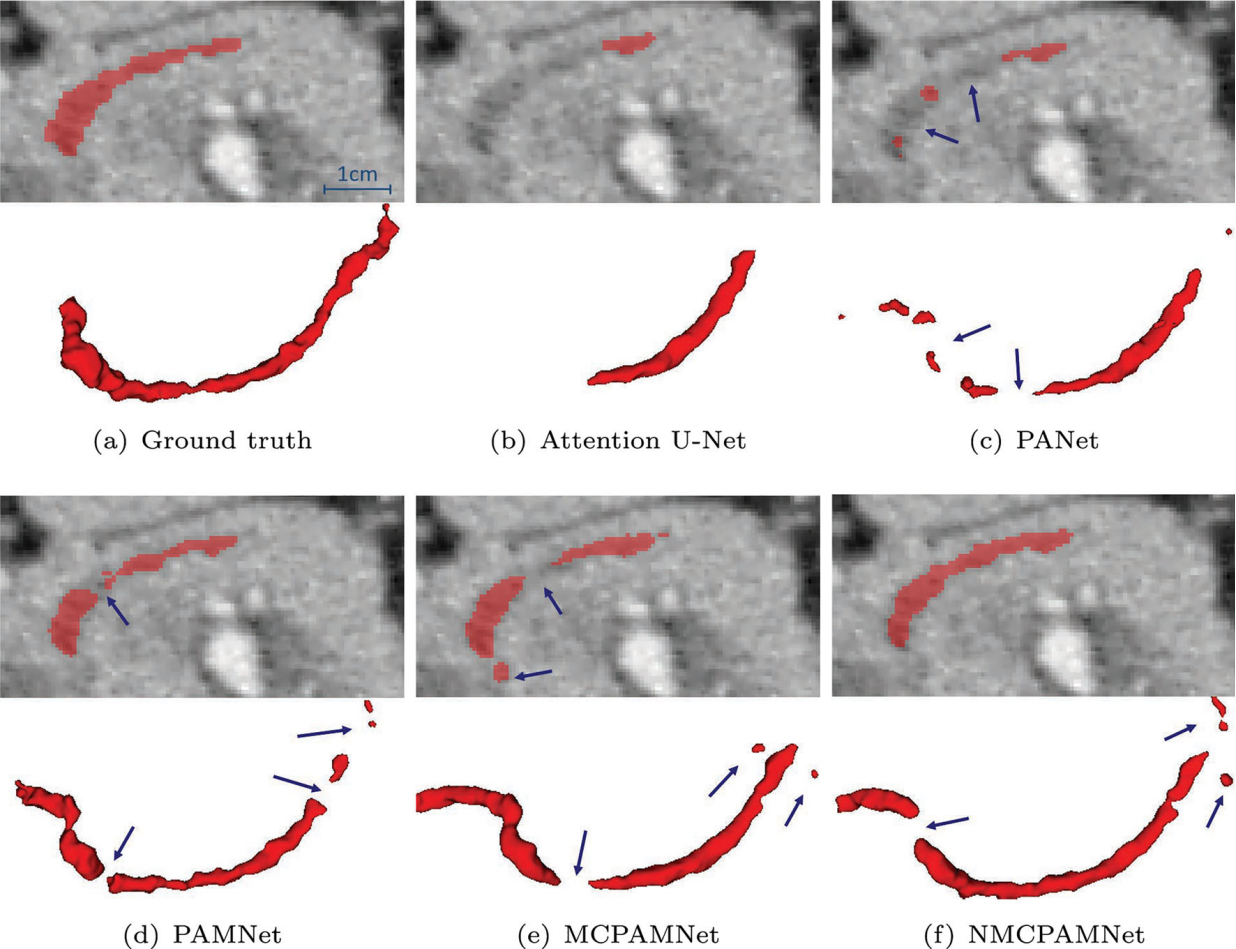
Comparison of **a** ground truth and pancreatic duct segmentation result using **b** Attention U-Net [11] **c** PANet, **d** PAMNet, **e** MCPAMNet, **f** NMCPAMNet. All the approaches here are using U-Net and BN as backbone. The segmentation failure is indicated by a blue arrow

**Fig. 5:**
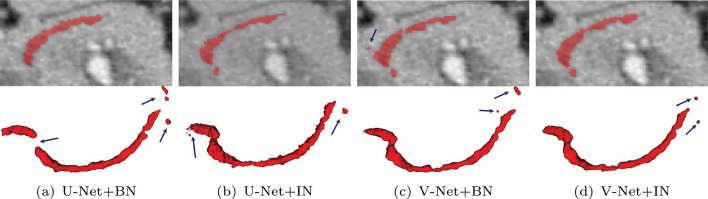
Comparison of pancreatic duct segmentation result using NMCPAMNet architecture with four different backbone settings: **a** U-Net+BN **b** U-Net+IN **c** V-Net+BN, and **d** V-Net+IN. The segmentation failure is indicated by a blue arrow. The ground truth of this case is shown in Fig. 4a

**Fig. 6:**
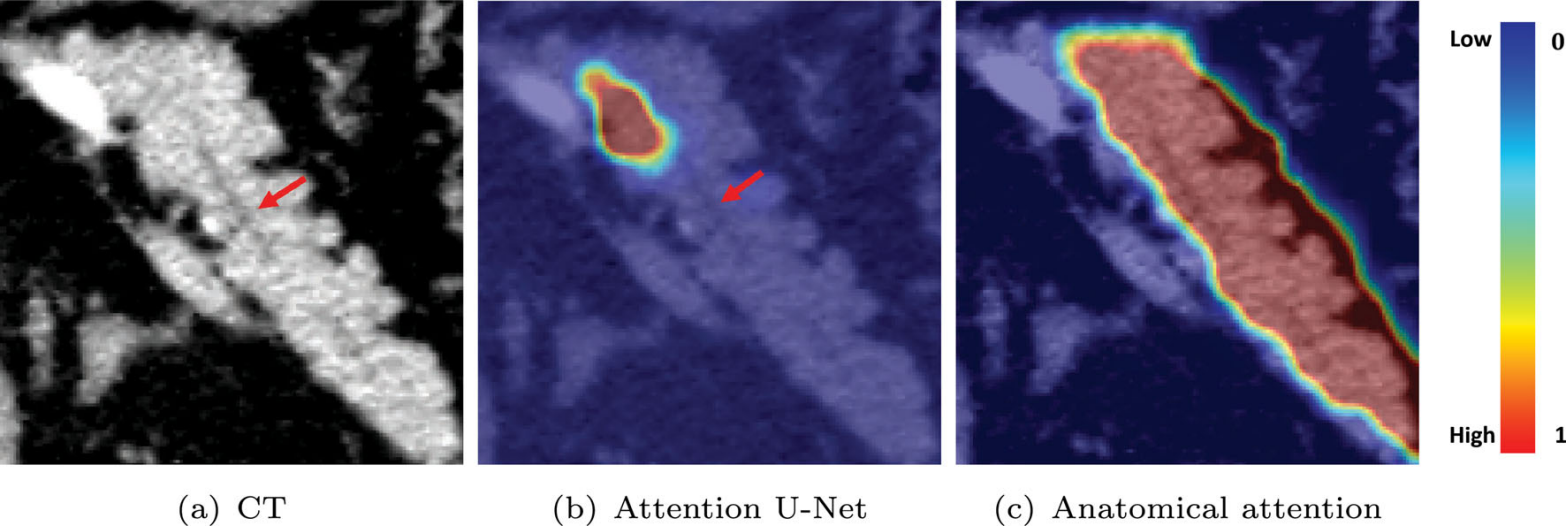
Heatmap visualization of attention coefficients on **a** CT using **b** Attention U-Net [11] and our proposed **c** Anatomical attention. The pancreatic duct is indicated by the red arrow inside the pancreas

Table 3 shows an ablation study of our proposed FCNs on the dilated pancreatic duct segmentation. Four metrics including Dice similarity score (DSC), sensitivity, NSD, and 95% Hausdorff Distance (HD95) are employed. We compared the standard Attention U-Net [11] with our proposed pancreatic anatomical attention network (PANet), as well as PANet with multi-scale aggregation (PAMNet) and further evaluated PAMNet with non-normalized (MCPAMNet) and normalized (NMCPAMNet) tubular structure enhancement. In the context of pancreatic duct segmentation, our baseline model is the Attention U-Net, with 3D U-Net serving as the backbone FCN. Recognizing V-Net’s strong performance in pancreas segmentation, we conducted additional experiments using V-Net as the backbone. Furthermore, we conducted evaluations of different FCNs with both IN and BN to identify the optimal combination The best results are obtained by NMCPAMNet using the V-Net as FCN backbone and IN as the normalization operation. Figure 4 shows segmentation examples of coronal slice and 3D rendering in each method with U-Net and BN as backbone. In Fig. 5, we present a segmentation comparison using NMCPAMNet with four different backbone combinations: U-Net+BN, U-Net+IN, V-Net+BN, and V-Net+IN. Figure 6 shows a comparison of the heatmap depiction of attention coefficients using Attention U-Net and our proposed anatomical attention.

Table 4 provides additional comparisons between the method we proposed and other reported pancreatic duct segmentation strategies. We must point out that [6] was carried out on PDAC patients, whose pancreatic ducts are substantially larger than normal cases. These studies used a dataset of 239 cases, which was much greater than the 30 examples we used. Our dataset was the same as in [10] and [15]. Although it is difficult to directly compare studies using different datasets, the approach we propose yields the highest DSC on pancreatic duct segmentation on single-phase CT volume.

## Discussion

For the pancreatic mask segmentation, increasing the input size and number of levels of the V-Net is both beneficial and efficient. On the other hand, increasing the initial filter number helps in some situations, but significantly raises the parameter numbers of FCN. Thus, it is necessary to strike a balance between segmentation performance and model complexity. For normalization, IN is better suited for pancreatic segmentation using V-Net than BN. When applying IN on V-Net, the DSC for pancreatic segmentation increased considerably at each network scale. Because BN performance is heavily dependent on batch size, which is limited by computer power.

**Table 4:** In comparison with previous pancreatic duct segmentation methods

Methods	Authors	Phase	# of data	DSC (%)
3D-UNet-single-phase [6]	Zhou et al.	Arterial	239 PDAC	38.35±28.98
3D-UNet-single-phase [6]	Zhou et al.	Portal venous	239 PDAC	40.25±27.89
3D-ResDSN-single-phase [6]	Zhou et al.	Arterial	239 PDAC	47.04±26.42
3D-ResDSN-single-phase [6]	Zhou et al.	Portal venous	239 PDAC	49.81±26.23
Cascade SE-Dense U-net [10]	Shen et al.	Portal venous	30 normal	49.87±22.54
MPA-Net [15]	Shen et al.	Portal venous	30 normal	54.16±12.60
**Proposed method**	**Shen et al.**	**Portal venous**	**30 normal**	**55**.**70**$\varvec{\pm }$**12**.**50**

Here we listed other segmentation results performed on single-phase CT volumes only. Our proposed methods are highlighted in bold

For pancreatic duct segmentation, focusing on the entire pancreas anatomy improves the segmentation compared to the original Attention U-Net [11]. In medical image analysis, it is not always optimal to focus on a particular target. Narrowing the FCN focus would result in a lower fault tolerance during training when the target region is quite small. This hypothesis was further affirmed by the visualization of attention coefficients in Fig. 6. Some pancreatic duct parts are outside of the focus in standard attention U-Net. The segmentation performance is also enhanced by multiscale aggregation of FCN, which makes full use of the knowledge acquired at each level. The DSC on pancreatic duct segmentation was significantly enhanced by introducing the normalized tubular structure enhancement as a second input channel. The additional channel helps FCN in understanding the duct’s tubular structure better. Our proposed NMCPAMNet with V-Net baseline and IN demonstrates the most favorable performance across all four metrics. It achieves the highest scores in DSC and lowest in HD95. While it may not have the highest accuracy in terms of sensitivity and NSD, its performance remains comparable to other methods. Segmentation examples of 3D rendering and coronal slice segmentation are shown in Fig. 4 and Fig. 5. The tubular structure enhancement can improve the connection of the pancreatic duct segmentation. The pancreatic duct segmented by NMCPAMNet using V-Net+IN as the backbone exhibits smoother duct segmentation with less exceeding segmentations.

We also compared our proposed method to existing pancreatic duct segmentation strategies that are published in Table 4. When compared to other reported results of pancreatic duct segmentation using single-phase CT volumes only, our method outperforms all existing strategies, despite the fact that we employed only 30 cases instead of the larger dataset’s 239 cases.

## Conclusions

We investigated an anatomical attention-based strategy for the segmentation of the dilated pancreatic duct from CT volumes. Our strategy was motivated by a usual clinical experience. When radiologists look for the pancreatic duct from the CT volumes, they first try to locate the pancreas area. We proposed an attention mechanism that enables to focus on the entire pancreas anatomy rather than just the target. To fully capture the vessel-like structure of the pancreatic duct, we employed a tubular structure enhancement as an additional input channel for our FCN. We evaluated our proposed FCNs using four different assessment measures, which demonstrated the effectiveness of our proposed method. Upon comparing our results with other reported results for pancreatic duct segmentation, our method exhibits significant superiority over other strategies that rely on single-phase CT volumes. Our technique might be applied to other tube-like structure segmentation tasks for other anatomies in the future. Nevertheless, the duct component still has some exceeding and improper segmentation. For the use of PDAC diagnosis in real-world settings, the overall accuracy still needs to be increased to capture the full anatomy of the duct. This remains as future work.
